# Bezoar in gastro-jejunostomy presenting with symptoms of gastric outlet obstruction: a case report and review of the literature

**DOI:** 10.1186/1752-1947-2-323

**Published:** 2008-10-02

**Authors:** Edmund Leung, Ruth Barnes, Ling Wong

**Affiliations:** 1 Department of Surgery, University Hospitals Coventry and Warwickshire, Coventry, CV2 2DX, UK

## Abstract

**Introduction:**

Gastric outlet obstruction usually presents with non-bilious vomiting, colicky epigastric pain, loss of appetite and occasionally, upper gastrointestinal bleeding. Causes can be classified as benign or malignant, or as extra- or intraluminal. Gastrojejunostomy is a well-recognised surgical procedure performed to bypass gastric outlet obstruction. A bezoar occurs most commonly in patients with impaired gastrointestinal motility or with a history of gastric surgery. It is an intestinal concretion, which fails to pass along the alimentary canal.

**Case presentation:**

A 62-year-old Asian woman with a history of gastrojejunostomy for peptic ulcer disease was admitted to hospital with epigastric pain, vomiting and dehydration. All investigations concluded gastric outlet obstruction secondary to a "stricture" at the site of gastrojejunostomy. Subsequent laparotomy revealed that the cause of the obstruction was a bezoar.

**Conclusion:**

Many bezoars can be removed endoscopically, but some will require operative intervention. Once removed, emphasis must be placed upon prevention of recurrence. Surgeons must learn to recognise and classify bezoars in order to provide the most effective therapy.

## Introduction

Gastric outlet obstruction (GOO) in adults is not a single entity; it is the pathophysiological consequence of any disease process that produces a mechanical impediment to gastric emptying. There are benign and malignant causes. In the past, peptic ulcer disease was more prevalent than malignant causes, currently, it only accounts for 5% of all cases of GOO [1]. With the advent of proton pump inhibitors and *Helicobacter pylori *eradication therapy, this benign cause has become less common. Andersson and Bergdahl reported [2] that 67% of patients have GOO secondary to malignancy. Other benign intraluminal causes in adults include gastric polyps, caustic ingestion, gallstone obstruction (Bouveret syndrome), and bezoars.

Bezoars, concretions of indigestible material in the gastrointestinal tract, have been known to occur in animals for centuries. The incidence of bezoars in adult patients has increased as a result of operative manipulation of the gastrointestinal tract. Although bezoars are often recognised radiologically, endoscopy provides the most accurate means of identification. Many bezoars can be removed endoscopically, but some will require operative intervention. Once removed, emphasis must be placed upon prevention of recurrence. Surgeons must learn to recognise and classify bezoars in order to provide the most effective therapy.

We report a case of a 62-year-old Asian woman with a history of gastrojejunostomy, who was admitted to hospital with GOO secondary to a bezoar. We present the case, discuss management and review the literature.

## Case presentation

A 62-year-old Asian woman presented acutely to the emergency department with a 1-day history of colicky epigastric pain and postprandial vomiting. She had been tolerating only liquids rather than solid food for 2 months. There was no history of weight loss, but she did report early satiety and loss of appetite.

**Figure 1:**
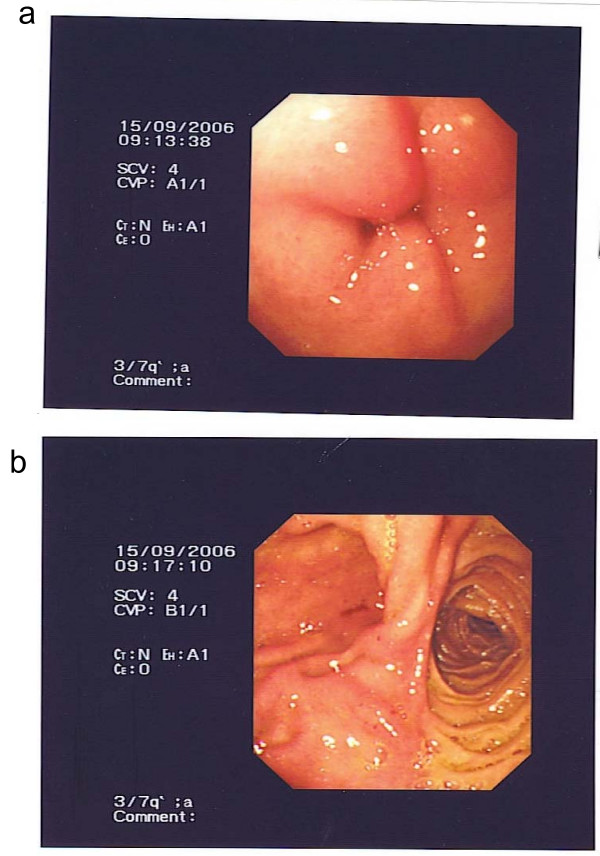
**Image taken during upper endoscopy**. a) Oedema present at the anastomotic site of the gastrojejunostomy. b) No evidence of obstruction beyond the anastomosis.

This woman had a history of peptic ulcer disease over 20 years ago in Kenya. It had led to GOO requiring truncal vagotomy and gastrojejunostomy. In order to investigate the cause of her dysphagia and loss of appetite, she had undergone an upper gastrointestinal endoscopy 3 weeks before this admission. This showed inflammation and oedema at the anastomotic site of the gastrojejunostomy, but no evidence of obstruction or stricture (Figure 1). She was then prescribed daily omeprazole, which was the only medication she was taking on admission.

The patient was clinically dehydrated on examination. She had a very thin body habitus. Her abdomen was soft, but mildly tender over her epigastrium. Succussion splash was demonstrated and a 10 cm × 8 cm mass was palpable just right of the umbilicus. Bowel sounds were scanty. There were no clinical signs for upper gastrointestinal bleeding.

**Figure 2:**
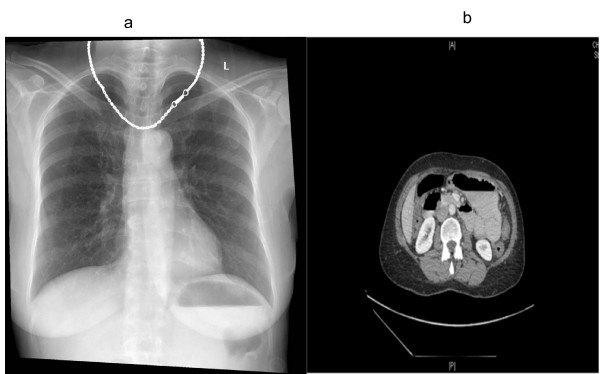
**Imaging**. a) Erect chest radiograph showing an air-fluid level within a dilated stomach. Lung fields were clear. There is no air under the diaphragm. b) Contrasted abdominal computed tomography showed possible stricture at the site of the gastrojejunostomy.

Her admission blood profiles were essentially unremarkable. There was no biochemical evidence of fluid shifts or dehydration. Plain abdominal radiograph did not show any diagnostic features. However, her erect chest radiograph showed an air-fluid level within a dilated stomach (Figure 2a).

In view of the examination and chest radiograph findings, she had a nasogastric tube and urinary catheter inserted for gastric decompression and urine output monitoring, respectively. An urgent contrasted computed tomography of the abdomen was arranged. Meanwhile, the nasogastric tube successfully prevented further vomiting, and there was little drainage from it. She was commenced on intravenous omeprazole and fluid therapy.

The abdominal computed tomography (Figure 2b) showed a fluid filled, non-dilated stomach. The anastomosis between the proximal jejunum and body of the stomach was shown to be patent. The afferent loop was not dilated but the efferent loop was dilated. Just past the midline, approximately 20 cm from the anastomotic site, there was a change in calibre of the bowel with the jejunum becoming significantly narrowed. The bowel distal to this site was collapsed. The proposed diagnosis was a stricture at the site of the gastrojejunostomy, but the exact cause was uncertain.

**Figure 3:**
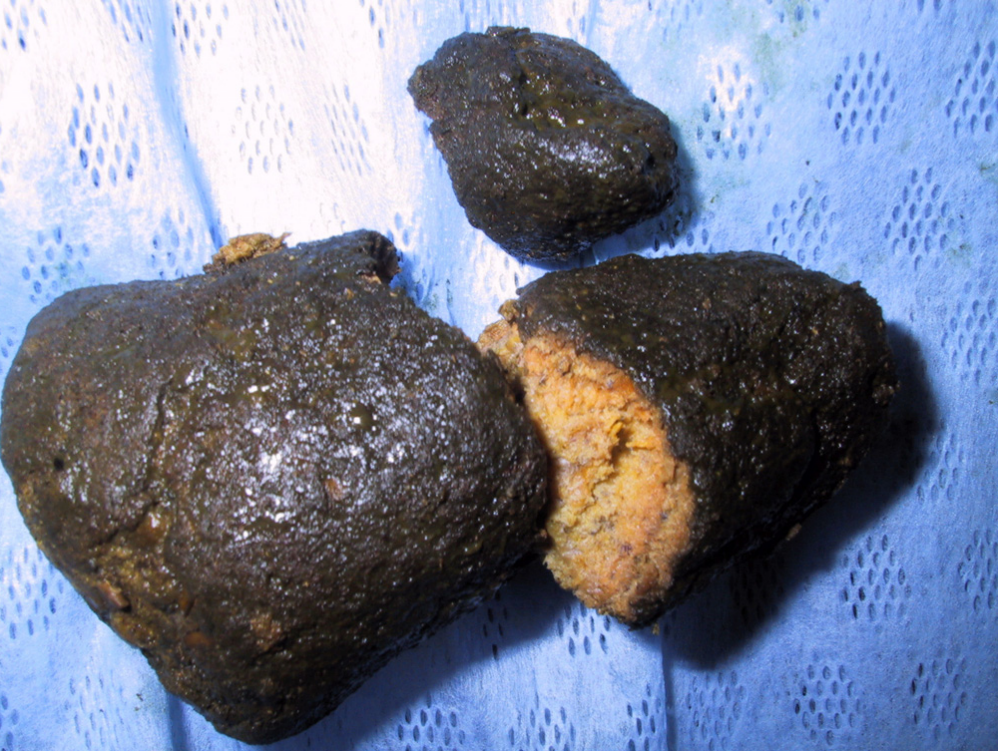
**A 10 cm conical phytobezoar was found 20 cm distal to the gastrojejunostomy.** It was removed by an enterotomy.

The patient provided consent for expedited laparotomy and relief of obstruction. Intra-operatively, the jejunum was found to be dilated from the duodenojejunal flexure to a large bolus obstruction. A conical mass suspicious of a bezoar was found measuring 10 cm in length, situated 20 cm beyond the gastrojejunostomy. The small bowel distal to this site was collapsed. Attempts to break up this hard bolus mass externally were unsuccessful. The bezoar eventually had to be removed in whole via an enterotomy. Careful examination confirmed that it was indeed a phytobezoar (Figure 3).

The patient had an uneventful recovery and was discharged home 1 week after surgery. Before discharge, she was seen by the dietician with regard to different types of fibre diet. She was also advised on the importance of longer mastication of food.

## Discussion

A bezoar is also known as an enterolith, a concretion of foreign or indigestible matter found in the alimentary canal. There are two main types of bezoars: trichobezoar – a bezoar formed from hair and phytobezoar – formed by indigestible cellulose. Rarely, pharmacobezoars from masses of tablets are found.

This was an unusual presentation of symptoms and signs of GOO secondary to a phytobezoar, in that this woman had already had a gastrojejunostomy to bypass previous GOO caused by peptic ulcer disease. The oedema seen in her upper endoscopy 3 weeks before admission may have been the result of a distal subacute obstruction. Postprandial non-bilious vomiting is the cardinal symptom of GOO, which may lead to electrolyte abnormalities. The frequency of vomiting puts patients at risk of aspiration pneumonia. Early satiety and better tolerance to liquid than solid food may represent gastric dilatation, which may be appreciated by succussion splash. Management includes identification of the cause and reversal of any complications of GOO such as metabolic alkalosis, electrolyte abnormalities, and aspiration pneumonia. Diagnosis can result from upper endoscopy or imaging studies.

Regardless of the cause, 75% of all cases of GOO require surgical intervention [3]. Definitive treatment consists of laparotomy with milking of the contents to the caecum, or enterotomy. Medical treatment is usually inadequate. Recently, the laparoscopic approach has become increasingly popular. A recent study compared laparoscopic versus open treatment for bezoar-induced small bowel obstruction [4]. The report concluded that laparoscopy is safe and effective and is associated with a better postoperative outcome and a shorter hospital stay. One author describes how a jejunal bezoar in a 59-year-old man was laparoscopically milked into the caecum through the ileocaecal valve [5].

Bezoars tend to be rare, except in patients with previous gastric surgery [6] or gastrointestinal dysmotility. In a 10-year retrospective review of all patients with small bowel obstruction in a hospital in Hong Kong, the incidence of bezoar was reported as approximately 2% [7]. A 4-year study in an Italian unit confirmed a similar incidence with nine of 369 patients having bowel obstruction secondary to bezoars [8]. It appears that geographical or dietary variation does not participate in the risk of developing bezoar obstruction.

Delayed gastric emptying and abnormal gastric motility patterns were prominent in one series of patients with bezoars, suggesting that these events were the underlying factors [9]. There was another series of patients with bezoar obstruction, who had had pyloroplasty for peptic ulcer disease. These patients did not demonstrate delayed gastric emptying when assessed by technetium-99m-labelled studies [10]. However, Cifuentes *et al. *[11] reported that 84% of cases of bezoar obstruction occurred in those who had had a bilateral truncal vagotomy and pyloroplasty. The authors proposed that, in this acid reducing procedure, there is hypochlorhydria, which reduces gastric antral motility and gives poor degradation of food. This predisposes to the formation of a ball of sticky concretions, which pass into the duodenum and jejunum unfragmented.

More evidence has since emerged supporting this theory. Another study [12] involving 117 patients with gastrointestinal bezoars revealed that 87% occurred in the small bowel and 30% in the stomach. Furthermore, 70% of patients had previous surgery for peptic ulcer disease, and 80% of these patients had a bilateral truncal vagotomy with pyloroplasty. Of the 87 patients presenting with intestinal bezoars, excessive intake of dietary fibre occurred in 40%, and 24% had alterations of mastication and dentition. There are other risk factors for bezoar obstruction. Children themselves are at higher risk than adults in that they have smaller gastrointestinal lumens, especially with trichobezoar obstruction. There is also an association between bezoar obstruction and mentally retarded patients [13].

As discussed, patients with bezoars often present with symptoms and clinical or radiological signs of bowel obstruction. Dilated small bowel loops may be seen in plain abdominal radiographs. In one retrospective study, the abdominal computed tomography scan was declared to be the most useful imaging modality for detecting bezoars [14]. The study advocated that abdominal computed tomography should be performed early in patients at higher risk of developing bezoars. The classical appearance of a bezoar on computed tomography is a well-defined ovoid intraluminal mass with mottled gas pattern at the site of obstruction.

Besides obstruction and its associated complications, other complications of bezoars include ulceration, intussusception, and bowel perforation. Intraluminal bezoar is a serious condition, with a mortality rate as high as 30% being reported in a retrospective analysis of 34 cases [15]. Early diagnosis and aggressive treatment is the key to successful management of the condition, which is curable.

## Conclusion

Bezoar induced bowel obstruction is uncommon and remains a diagnostic challenge. It should be suspected in patients with an increased risk, such as those with previous gastric surgery, poor dentition, mental retardation and a suggestive history of increased fibre intake. Computed tomography of the abdomen should be performed early in these at-risk patients presenting with symptoms of GOO or small bowel obstruction in order to reduce unnecessary delays before appropriate surgical intervention. Bezoar is a curable condition but can potentially cause significant morbidity and mortality.

## Consent

Written informed consent was obtained from the patient for publication of this case report and any accompanying images. A copy of the written consent is available for review by the Editor-in-Chief of this journal.

## Competing interests

The authors declare that they have no competing interests.

## Authors' contributions

EL revised the manuscript and researched the case while LW supervised the ethical approval, patient consent, patient management and amendments to the manuscript. RB acquired patient information, designed and drafted the manuscript, sorted patient consent, carried out day-to-day management of patient and provided intellectual content to the discussion of this case report.
